# Relevance of HLA-DP/DQ and ICAM-1 SNPs among Ovarian Cancer Patients

**DOI:** 10.3389/fimmu.2016.00202

**Published:** 2016-05-24

**Authors:** Amany A. Ghazy, Nour M. El-Etreby

**Affiliations:** ^1^Department of Immunology and Allergy, Medical Research Institute, Alexandria University, Alexandria, Egypt; ^2^Obstetrics and Gynecology Department, Faculty of Medicine, Alexandria University, Alexandria, Egypt

**Keywords:** ovarian cancer, HLA-DP, HLA-DQ, SNP, ICAM-1

## Abstract

**Background:**

Ovarian cancer is one of the most lethal gynecological malignancies and the fifth leading cause of cancer deaths among women. The high mortality rate is largely attributed to its diagnosis in advanced stages. Poor prognosis of ovarian cancer is usually due to the lack of specific or effective screening and diagnostic methods for identifying early-stage disease.

**Aim:**

Our study aimed to study the role of HLA-DP, HLA-DQ, and ICAM-1 SNPs in diagnosis and/or prognosis of ovarian tumors

**Subjects and methods:**

The current study was conducted on 60 patients with ovarian tumors (benign, borderline, and malignant) and 20 healthy volunteers. Genotyping of HLA-DP rs3077, HLA-DQ rs3920, and ICAM-1 rs1437 SNPs was done using 5′ nuclease assay.

**Results:**

We found significant association of HLA-DP rs3077 AA, HLA-DQ rs3920 GG, ICAM-1 rs1437 CC, and CT genotypes with increased risk of ovarian cancer (OR = 43.5, 6, 25, and 2.6, respectively). In addition, HLA-DQ rs3920 and ICAM-1 rs1437 alleles vary significantly among different types of ovarian cancer (*P* = 0.003 and 0.001, respectively).

**Conclusion:**

HLA-DP rs3077, HLA-DQ rs3920, and ICAM-1 rs1437 SNPs could help in the diagnosis and prognosis of ovarian cancer.

## Background

Ovarian cancer is considered the most lethal gynecological malignancies and the fifth leading cause of cancer deaths among women, according to the National Cancer Institute’s (NCI) 2014 statistics (1, 2). Majority of the cases are diagnosed in the late stages of the disease, resulting in poor survival (3). The 5-year survival rate of patients with ovarian cancer is only around 30% in Stage III or IV (4). This high mortality rate is attributed to the fact that ovarian cancer develops without obvious symptoms resulting in advanced and widespread disease when patients are first diagnosed (5).

Ovarian cancer has three main types; epithelial ovarian cancer (EOC), stromal tumors, and germ cell tumors. EOC represents about 90% of ovarian cancers and is classified into serous, mucinous, endometrioid, clear cell, and transitional cell tumors (2). Globally, EOC is the leading cause of mortality among gynecological malignancies. Therefore, developing novel predictive biomarkers for recurrence and metastasis is urgently needed to improve the survival of patients with EOC (6).

Several independent prognostic factors including age, performance status, FIGO stage, grade of tumor, and volume of residual tumor have been established in predicting survival in ovarian cancer patients. However, these macro factors are insufficient to predict the outcomes. Hence, it is necessary to identify new prognostic molecular factors to predict the outcomes of patients and select the suitable treatment options for ovarian cancer patients (7).

During the past few years, accumulating evidences support the hypothesis that molecular events may be responsible for tumor initiation, metastasis, recurrence, and resistance to treatment.

Human leukocyte antigens (HLAs) are very important in tumor surveillance, since they can modulate the efficacy of cytotoxic T-lymphocytes through the presentation of tumor antigens. In some ovarian cancers, tumor-infiltrating lymphocytes (TILs) have been identified, indicating a local immune response and better prognosis. It is believed that HLA-class II antigens influence the interaction between ovarian cancer cells and the host’s immune response, as an increase in HLA-class II molecules on tumor cells will augment the existence of TILs (8). But, molecular mechanisms responsible for altered HLA-class II phenotypes in ovarian tumors need further research.

Moreover, intercellular adhesion molecule-1 (ICAM-1 CD54) is an inducible cell-surface glycoprotein from immunoglobulin supergene family, which is essential for most cell–cell interactions in the immune response (9). It has a role in tumorigenesis and tumor progression, as it modulates the occurrence and prognosis of several types of cancers (10); however, its role in EOC remains unclear.

Thus, this study aimed to investigate the role of HLA-DP rs3077 (A/G), HLA-DQ rs3920 (A/G), and ICAM-1 rs1437 (C/T) SNPs in diagnosis and/or prognosis of ovarian cancer.

## Subjects

This study was conducted on 80 subjects; 60 patients with ovarian tumors from Obstetrics and Gynecology Department, Faculty of Medicine, Alexandria University and 20 healthy volunteers. Subjects were divided into four groups; group 1 included 20 patients with benign lesions (serous cyst and cystadenofibroma); 7 females were at menopause, while 13 were in pre-menopause state, group 2 included 20 patients with borderline ovarian cancer (15 serous and 5 mucinous); 12 females were at menopause, while 8 were in pre-menopause state, group 3 included 20 patients with malignant ovarian cancer (6 serous, 4 mucinous, 7 endometrioid, and 3 poorly/undifferentiated); 10 females were at menopause, while 10 were in pre-menopause state, and group 4 (control group) age-matched healthy females; 4 were at menopause, while 16 were in pre-menopause state.

All participants were asked to freely volunteer to the study, and informed written consents were gathered prior to their inclusion in the study protocol, according to ethical guidelines of the Medical Research Institute, Alexandria University (Informed Written Consent for Patient Participation in a Clinical Research, 2011).

## Materials and Methods

All reagents used in the study were supplied by Applied Biosystems, Life Technology Company (St. Louis, MO, USA).

Assessments of HLA-DP rs3077 (A/G), HLA-DQ rs3920 (A/G), and ICAM-1 rs1437 (C/T) SNPs were performed following the instructions in TaqMan^®^ SNP Genotyping Assays Protocol provided by Applied Biosystems, Life Technology Company. Genomic DNA was extracted from whole blood samples in EDTA vacutainers using the PureLink^®^ Genomic DNA Kits # K1820-01 (Invitrogen, Life Technologies) followed by assessment of DNA concentration and purity using a Nanodrop spectrophotometer. The extracted DNA was stored at −80°C till used.

HLA-DP rs3077 (A/G), HLA-DQ rs3920 (A/G), and ICAM-1 rs1437 (C/T) polymorphisms were analyzed using 5′ nuclease assay with a TaqMan MGB (minor groove binder) probe in a *StepOne*™ Real-Time PCR System (Applied Biosystems, Life Technologies). SNP Genotyping Assays contain VIC^®^ dye-labeled probe, FAM™ dye-labeled probe, and two target-specific primers. TaqMan^®^ probes incorporate MGB technology at the 3′-end to deliver superior allelic discrimination. All MGB probes also include a non-fluorescent quencher (NFQ) that virtually eliminates the background fluorescence and provides excellent signal-to-noise ratio for superior assay sensitivity.

HLA-DP rs3077, HLA-DQ rs3920, and ICAM-1 rs1437 primers and TaqMan MGB probes were provided by the assay on-demand™ service by Applied Biosystems, Life Technologies. 2 μl genomic DNA, 7 μl DNase-free water, 10 μl TaqMan Universal PCR Master Mix (2×), and 1 μl working stock of SNP genotyping assay (20×) were added in PCR tubes. The assay contain sequence-specific forward and reverse primers to amplify the polymorphic sequence of interest and two TaqMan^®^ MGB probes with NFQ (one VIC^®^-labeled probe to detect Allele 1 sequence and one FAM™-labeled probe to detect Allele 2 sequence). Negative controls (without DNA samples) were included in each run. Thermal cycling conditions were adjusted to be 10 min at 95°C, followed by 40 PCR cycles each consisting of 15 s at 92°C and 1 min at 60°C. Each reaction plate was loaded into *StepOne*™ Real-Time PCR System, and then the run started.

**Figure 1:**
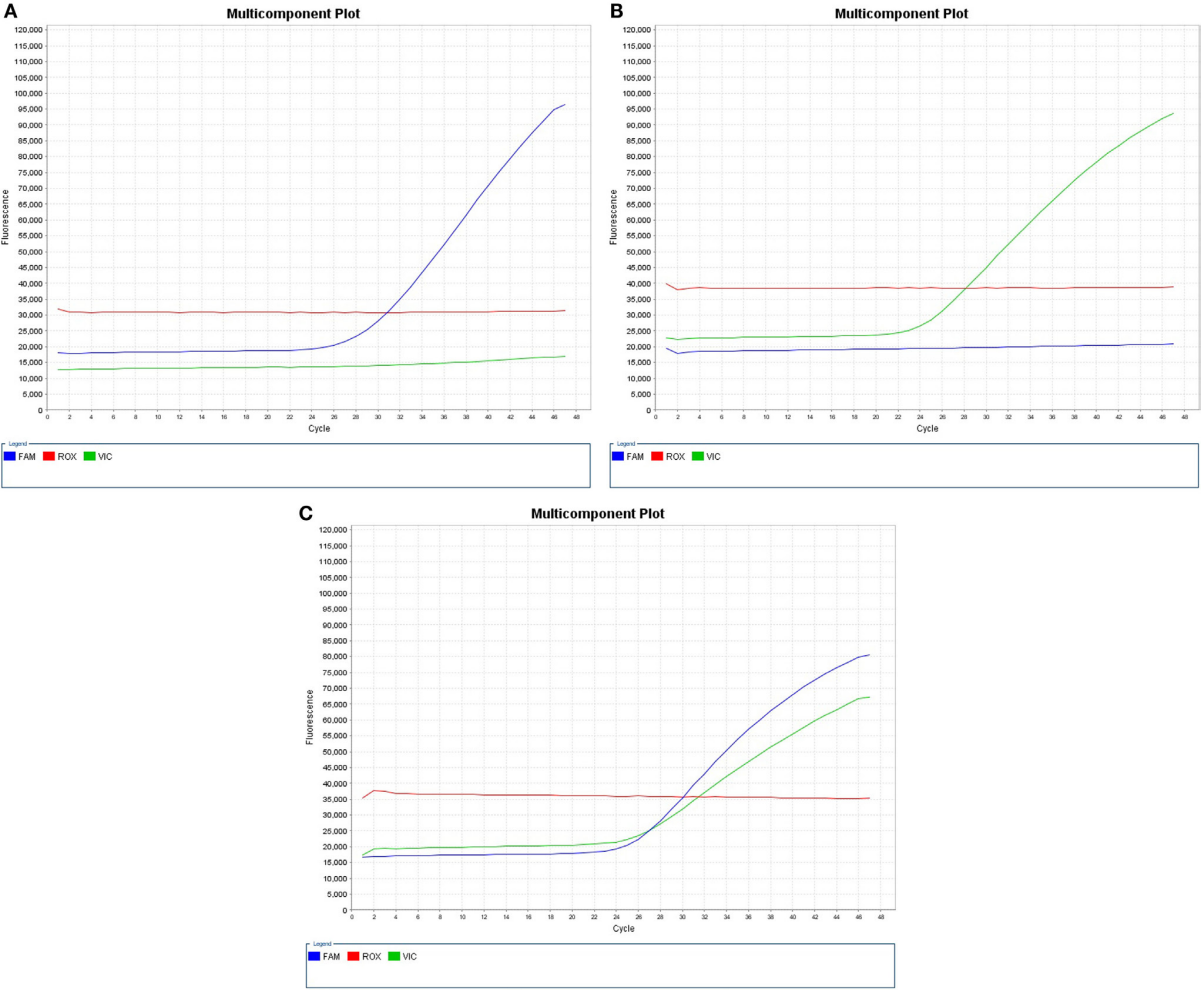
**Real-time PCR pictures displaying (A) expression of HLA-DP rs3077GG, HLA-DQ rs3920 GG, or ICAM-1 rs1437 TT, (B) expression of HLA-DP rs3077AA, HLA-DQ rs3920 AA, or ICAM-1 rs1437 CC, and (C) heterozygous expression of both alleles “HLA-DP rs3077AG, HLA-DQ rs3920 AG, or ICAM-1 rs1437 CT**.”

When probes hybridized to the complementary sequence are cleaved, an increase in fluorescence signal occurs. Thus, the fluorescence signal generated by PCR amplification indicates which alleles are present in the sample (Table 1; Figure 1). In our study, VIC^®^ dye is associated with the A allele of HLA-DP rs3077, A allele of HLA-DQ rs3920, and C allele of ICAM-1 rs1437, while FAM™ dye is associated with the G allele of HLA-DP rs3077, G allele of HLA-DQ rs3920, and T allele of ICAM-1 rs1437.

**Table 1:** **Correlation between fluorescence signals and allele present in a sample**.

**A substantial increase in…**	**Indicates…**
VIC-dye fluorescence only	Homozygous for allele 1
FAM-dye fluorescence only	Homozygous for allele 2
Both VIC- and FAM-dye fluorescence	Allele 1–allele-2 heterozygous

### Statistical Analysis of the Data

Data were analyzed using IBM SPSS software package version 20.0. Qualitative data were described using number and percentage. Comparison between different groups regarding categorical variables was tested using Chi-square test. When more than 20% of the cells have expected count less than five, correction for Chi-square was conducted using Monte Carlo correction. Significance of the obtained results was judged at the 5% level (11).

## Results

### Subject’s Demographic Data

Age distributions among benign, borderline, and malignant ovarian tumors and healthy control group did not show any statistically significant differences. While regarding menstrual state, there were statistically significant differences between borderline and malignant patients with the other groups (Table 2).

**Table 2:** **Distribution of age and menstrual state in the studied groups**.

**Characteristics**	**Group**								***MCP***
	**Benign**		**Borderline**		**Malignant**		**Control**		
	**No**	**%**	**No**	**%**	**No**	**%**	**No**	**%**	
**Age (years)**									
• <40	4	20.0	4	20.0	3	15.0	9	45.0	0.06
• 40–50	6	30.0	4	20.0	3	15.0	5	25.0	
• 50–60	6	30.0	8	40.0	6	30.0	4	20.0	
• 60–70	4	20.0	4	20.0	8	40.0	2	10.0	
**Menstrual status**									
• Pre-menopause	13	65.0	8	40.0	10	50.0	16	80.0	0.047*
• Menopause	7	35.0	12	60.0	10	50.0	4	20.0	

*MCP, Mont Carlo exact probability*.

*P < 0.05 (significant).

### HLA-DP rs3077 Variants among the Studied Groups

HLA-DP rs3077 AA genotype was expressed in 90, 100, 100, and 40% of benign, borderline, malignant ovarian tumors, and healthy volunteers, respectively, while AG genotype was expressed in 10, 0, 0, and 60% of benign, borderline, malignant ovarian tumors, and healthy volunteers, respectively. There were no any statistically significant differences among the studied groups (*P* = 0.126) (Table 3).

**Table 3:** **Distribution of HLA-DP-rs3077, HLA-DQ-rs3920, and ICAM-1 rs1437 alleles in the studied groups**.

**Gene**	**Group**								***MCP***
	**Benign**		**Borderline**		**Malignant**		**Controls**		
	**No**	**%**	**No**	**%**	**No**	**%**	**No**	**%**	
**HLA-DP rs3077**									
• AA	18	90.0	20	100.0	20	100.0	8	40.0	0.126
• AG	2	10.0	0	0.0	0	0.0	12	60.0	
**HLA-DQ rs3920**									
• AG	2	10.0	12	60.0	6	30.0	15	75.0	0.003*
• GG	18	90.0	8	40.0	14	70.0	5	25.0	
**ICAM-1 rs1437**									
• CC	2	10.0	0	0.0	14	70.0	0	0.0	0.001*
• CT	7	35.0	16	80.0	5	25.0	8	40.0	
• TT	11	55.0	4	20.0	1	5.0	12	60.0	

*MCP, Mont Carlo exact probability*.

*P < 0.05 (significant).

**Figure 2:**
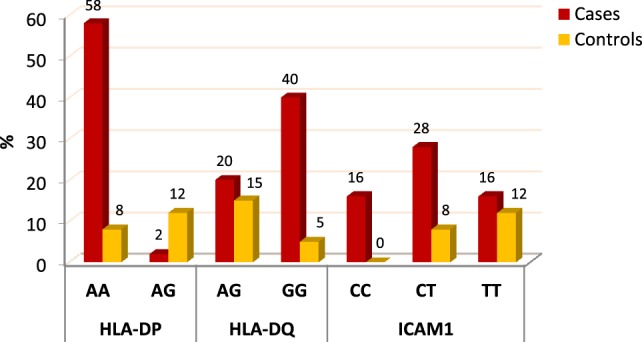
**Distribution of the studied alleles in ovarian tumors’ patients and healthy controls**.

When comparing between patients having ovarian tumors and control group, we found that HLA-DP rs3077 AA genotype was expressed in 96.7 and 40% of ovarian tumors patients and healthy volunteers, respectively, while AG genotype was expressed in 3.3 and 60% of ovarian tumors patients and healthy volunteers, respectively. There was statistically significant association between HLA-DP rs3077 AA genotype and the occurrence of ovarian cancer, as AA allele has 43.5-folds more odds to be cases compared to AG allele. So, HLA-DP rs3077 AA is a good predictor for ovarian cancer (OR = 43.5, 95% CI = 8.2–57.3) (Table 4; Figure 2). Correlation analysis clarified that HLA-DP rs3077 AA is highly expressed in all ovarian cancer females regardless the menstrual state (Table 5).

**Table 4:** **Distribution of the studied alleles in ovarian cancer patients and healthy controls**.

**Gene**	**Group**				**OR (95% CI)**
	**Cases**		**Controls**		
	**No**	**%**	**No**	**%**	
**HLA-DP rs3077**					
• AA	58	96.7	8	40.0	43.5 (8.2–57.3)*
• AG	2	3.3	12	60.0	1
**HLA-DQ rs3920**					
• AG	20	33.3	15	75.0	1
• GG	40	66.7	5	25.0	6.0 (1.9–18.8)*
**ICAM-1 rs1437**					
• CC	16	26.7	0	0.0	25.0 (1.4–125.6)*
• CT	28	46.7	8	40.0	2.6 (1.0–7.8)*
• TT	16	26.7	12	60.0	1

*OR, odds ratio; CI, confidence interval*.

*Significant (*P* < 0.05).

**Table 5:** **Relation between menstrual status and the studied alleles**.

**Menstrual status**	**Genotype**		**Group**				**MCP**
			**Cases**		**Controls**		
			**No**	**%**	**No**	**%**	
Pre-menopause
	HLA-DP	AA	29	93.5	4	25.0	0.001*
		AG	2	6.5	12	75.0	
	HLA-DQ	AG	7	22.6	11	68.8	0.002*
		GG	24	77.4	5	31.3	
	ICAM1	CC	8	25.8	0	0.0	0.067
		CT	11	35.5	6	37.5	
		TT	12	38.7	10	62.5	
Menopause
	HLA-DP	AA	29	100.0	4	100.0	–
		AG	0	0.0	0	0.0	
	HLA-DQ	AG	13	44.8	4	100.0	0.038*
		GG	16	55.2	0	0.0	
	ICAM1	CC	8	27.6	0	0.0	0.159
		CT	17	58.6	2	50.0	
		TT	4	13.8	2	50.0	

*Significant (*P* < 0.05).

### HLA-DQ rs3920 Variants among the Studied Groups

HLA-DQ rs3920 AG genotype was expressed in 10, 60, 30, and 75% of benign, borderline, malignant ovarian tumors, and healthy volunteers, respectively, while GG genotype was expressed in 90, 40, 70, and 25% of benign, borderline, malignant ovarian tumors, and healthy volunteers, respectively. There were statistically significant differences among the studied groups (*P* = 0.003), so HLA-DQ rs3920 alleles could be good predictors for type of ovarian tumor (Table 3).

When comparing between patients having ovarian tumors and control group, we found that HLA-DQ rs3920 AG genotype was expressed in 33.3 and 75% of ovarian tumors patients and healthy volunteers, respectively, while GG genotype was expressed in 40 and 25% of ovarian tumors patients and healthy volunteers, respectively. There was statistically significant relation between HLA-DQ rs3920 GG genotype and risk of ovarian cancer, as HLA-DQ rs3920 GG allele has sixfolds more odds to be cases compared to AG allele. So, HLA-DQ rs3920 GG is a good predictor for ovarian cancer (OR = 6, 95% CI = 1.9–18.8) (Table 4; Figure 2). Correlation analysis clarified that HLA-DQ rs3920 GG is highly expressed in all ovarian cancer females regardless the menstrual state (Table 5).

### ICAM-1 rs1437 Variants among the Studied Groups

ICAM-1 rs1437 CC genotype was expressed in 10, 0, 70, and 0% of benign, borderline, malignant ovarian tumors, and healthy volunteers, respectively, while CT genotype was expressed in 35, 80, 25, and 40% of benign, borderline, malignant ovarian tumors, and healthy volunteers, respectively. There were statistically significant differences among the studied groups (*P* = 0.001), so ICAM-1 rs1437 SNP could be a good predictor for progression of ovarian tumor (Table 3).

When comparing between patients having ovarian tumors and control group, we found that ICAM-1 rs1437 CC, CT, and TT genotypes were expressed in 26.7, 46.7, and 26.7% of ovarian tumors patients, respectively, while for healthy volunteers; CC, CT, and TT genotypes were expressed in 0, 40, and 60% of healthy volunteers, respectively, There was statistically significant association between ICAM-1 rs1437 CC and CT genotypes and ovarian cancer, as they have 25- and 2.6-folds, respectively, more odds to be ovarian cancer cases compared to TT allele. So ICAM-1 rs1437 CC and CT genotypes are good predictors for ovarian cancer; particularly CC allele (Table 4; Figure 2).

## Discussion

Ovarian cancer ranks among the top 10 diagnosed cancers and top 5 deadliest cancers in most countries (12). Prognosis of ovarian cancer is usually poor, due to the lack of effective screening and diagnostic methods to identify early-stage disease (7). Unfortunately, the molecular mechanisms of ovarian cancer remain unclear, and the attempts to develop effective methods that help in early detection and monitoring of tumor progression have great potential to improve patients’ survival. Thus in our samples, we have analyzed HLA-DP rs3077 (A/G), HLA-DQ rs3920 (A/G), and ICAM-1 rs281437 (C/T) SNPs in benign, borderline, malignant ovarian tumors’ patients, and normal controls.

We found that HLA-DP rs3077 AA and HLA-DQ rs3920 GG alleles are significantly associated with ovarian cancer incidence (OR = 43.5 and 6, respectively). Thus, they could help in early diagnosis of that disease. This can be explained by the fact that HLA-II presents tumor antigens to immune cells that are responsible for their clearance. Additionally, distribution of HLA-DQ rs3920 alleles varies greatly among different types of ovarian cancer (*P* = 0.003).

Expression of HLA-DP rs3077 A and/or HLA-DQ rs3920 G increases with the increase in tumor aggressiveness, as their expression was mild in control, moderate among benign, and high in borderline and malignant groups. This aberrant expression may have a role in tumor progression, advanced stages of tumor, and bad prognosis of patients.

These results were in agreement with Kübler et al. (8, 13), who have documented that MHC complex has a central role within the immunological integrity of the ovary, and the aberrant expression of HLA-class I and II molecules are supposed to contribute to cancer susceptibility or resistance to treatment. They recommended further investigations about the involvement of HLA-class II genes in the pathogenesis of this disease. Jiang et al. (14) have focused on the role of HLA in cervical cancer risk, and they found consistently significant associations of HLA-DP rs3077 with increased risks of cervical cancer (OR = 1.51, 95% CI = 1.32–1.71) and proposed it as susceptibility marker for cervical cancer among Chinese females. So, further validation studies on larger sample size are needed in our population.

Additionally, abnormal expression of adhesion molecules, as ICAM-1, has an integral role in tumor growth, invasion, metastasis, and evasion from the host immune defense (9). This agree with our results, where there was statistically significant association between ICAM-1 rs1437 CC and CT genotypes and ovarian cancer (OR = 25 and 2.6, respectively), so they are good predictors for ovarian cancer risk; particularly CC allele. Furthermore, ICAM-1 rs1437 alleles vary significantly among the different types of ovarian cancer where T allele is expressed more among benign cases, while C allele expression increases in the aggressive types (*P* = 0.001). So, they could help in predicting the disease progression and prognosis of ovarian cancer patients.

Cai et al. (10) have evaluated SNPs in ICAM-1 as predictors of EOC risk and prognosis. They found that ICAM-1 rs5498 G allele is associated with increased tumor grade (OR = 2.650) and risk (OR = 1.405). This risk was more evident in females who had first-degree relatives with a tumor (OR = 3.475) or who experienced early menarche (OR = 2.774). They concluded that ICAM-1 rs5498 likely confers a high risk for EOC in G allele carriers accompanied by upregulation of ICAM-1 expression during carcinogenesis. The combination of ICAM-1 rs5498 and tumor history predicts the EOC prognosis.

Indeed, HLA-class II and/or ICAM-1 SNP may influence the tumor-specific mechanisms, as the statistical analysis showed strong association between certain alleles and ovarian cancer; particularly aggressive types.

## Conclusion

The results of the present study provides a clue on the relevance of HLA-DP rs3077, HLA-DQ rs3920, and ICAM-1 rs1437 variants in ovarian cancer, as some alleles were identified to be linked with ovarian cancer as HLA-DP rs3077 AA, HLA-DQ rs3920 GG, ICAM-1 rs1437 CC, and CT genotypes (OR = 43.5, 6, 25, and 2.6, respectively). These alleles confer a high risk for EOC among their carriers’ especially first-degree relatives of patients. Additionally, we found that HLA-DQ rs3920 and ICAM-1 rs1437 alleles vary among different groups of ovarian cancer and are good predictors for tumor type (*P* = 0.003 and 0.001, respectively). They could help in predicting prognosis of ovarian cancer patients. The main limitation in our study is the small sample size. So, further research on a larger patient sample is recommended to confirm the positive correlation between ovarian cancer and these variants.

## Author Contributions

Dr. NE-E was responsible for collecting samples of patients and controls. Dr. AG was responsible for DNA extraction and detection of SNP using real-time PCR.

## Conflict of Interest Statement

The authors declare that the research was conducted in the absence of any commercial or financial relationships that could be construed as a potential conflict of interest.
